# Effect of Feed Intake on Water Consumption in Horses: Relevance to Maintenance Fluid Therapy

**DOI:** 10.3389/fvets.2021.626081

**Published:** 2021-03-01

**Authors:** David E. Freeman

**Affiliations:** Department of Large Animal Clinical Sciences, College of Veterinary Medicine, University of Florida, Gainesville, FL, United States

**Keywords:** horse, water consumption, feed intake, maintenance therapy, fluid therapy

## Abstract

Maintenance fluid therapy is challenging in horses that cannot drink or are denied feed and water because of concerns about gastrointestinal tract function and patency. Intravenous fluid delivery to meet water needs based on current recommendations for maintenance requirements were obtained in fed horses and therefore might not apply to horses that are not being fed. This is a critical flaw because of the interdependence between intestinal tract water and extracellular water to support digestion while preserving water balance, a concept explained by the enterosystemic cycle. Because horses drink less when they are not eating and hence have lower water needs than fed horses, maintenance water requirements need to be adjusted accordingly. This article reviews this topic and identifies benefits of adjusting maintenance fluid therapy to meet lower demands from gastrointestinal function, such as reduced volumes, lower cost, avoidance of overhydration.

Water is an essential nutrient for all mammals (1), especially the horse, a large herbivore with considerable water needs to support microbial fermentation in its voluminous hindgut. Water is generally provided to horses in two forms, as maintenance and replacement solutions. The latter involves intravenous infusion of large volumes of physiologic solutions with water and electrolyte composition similar to plasma, and is usually given to replace losses through disease (fluid resuscitation). Such fluid therapy is challenging because reliable guidelines for safe and effective infusion rates and volumes are poorly developed in veterinary medicine (2).

## Maintenance Rates of Water Intake

Maintenance fluid intake refers largely to water required to offset water losses through insensible routes (skin and lungs) and through excretory processes (urine and feces) (3–5). In healthy, euvolemic horses with a functional gastrointestinal tract, this is satisfied through voluntary water consumption, which is also used as the method to determine maintenance water requirements. Provision of maintenance fluids to horses with a nonfunctional gastrointestinal tract is more challenging and is prone to error (see below).

The widely accepted maintenance requirement of water for horses is approximately 60 ml/kg/day (2 to 3 ml/kg/h), or approximately 30 L/day for an average adult nonbreeding, nonworking horse (500 kg body weight) (5–7). In addition to consumed water, water is also provided in the feed, which could contribute ~3 ml/kg/day or ~1.6 L/horse/day (8, 9). Added to this is unmeasured water from metabolic oxidation, ~5 ml/kg/day (8, 9). When these sources are included, horses consume 62.4 to 71.4 ml/kg/day (6, 8–11). Water excretion through feces can be 2.5 to almost 3 times mean urine volume (9), although the opposite relationship has also been recorded, explained by variations in ration composition and between study subjects (10). Insensible loss of water through skin and lungs could be estimated at 5.2 to 16.8 L/500 kg/day (9–11), with higher volumes in response to activity and high ambient temperatures (12–14). Visible sweating is not required to produce evaporative losses of water and electrolytes through skin (10).

Water needs increase with increased salt and protein intake, dietary effects (7), forage intake (15), transportation (16–18), athletic activities (12, 13), and fever (14). Lactating mares can increase water consumption by 37 to 74% above maintenance needs to meet milk production (19, 20). Foals, especially neonates, also handle water differently compared to adults, and this needs to be factored into their fluid therapy, and they are at risk of overhydration because of their small size (21). Healthy neonatal foals <24 h old have larger fluid compartments than adult horses and have an almost fetal system for fluid distribution that allows fluid movement from a large interstitial fluid space to preserve intravascular volume. Whereas, adults retain 20 to 50% of IV isotonic fluids in the intravascular space for 30 to 60 min after infusion, intravascular retention in the neonate is only 6 to 7% of the infused volume (21). This can be explained by the high capillary filtration coefficient that causes a rapid fluid movement into the interstitial fluid in response to a transient increase in capillary pressure (21). The fetal interstitium contains more ground substance and is quite compliant, so it can hold large amounts of fluid without generating increased interstitial pressure or edema (21).

Feeding schedules (22, 23) and dietary management (24) can profoundly influence maintenance water consumption in horses, which suggests that water intake is regulated to support feed consumption. More digestible hay-grain diets induce lower water consumption than hay only, which has been attributed to the lower needs for water to aid excretion of the smaller intestinal bulk (7). High dry matter content and cell wall constituents in the diet also increase water intake (7). Legume forages high in protein, such as alfalfa, require as much water as grass forages but induce a greater urinary excretion of water (7, 8). In horses transitioned from pasture to stall management with controlled exercise, water consumption doubled on all recorded days after the transition, total fecal output decreased by about half, and fecal dry matter content increased compared with pasture measurements (24). These changes in intestinal water, along with decreased motility in different parts of the large colon (on ultrasound examination), could be implicated as possible causes of colonic impactions (24).

## Effect of Feed Intake on Water Consumption

Maintenance fluid requirements have been determined on fed horses, and so might not be clinically relevant to horses denied feed, are unable to eat, or are unwilling to eat as part of their response to their primary disease and its treatment. Other examples are healthy euvolemic horses deprived of feed for sporting activities (17), transport (12, 16), and general anesthesia (25), or are anorexic or denied feed for any reason (6, 26–28). Maintenance water needs in horses can be influenced by feed intake, largely through the role of water in the extracellular fluid (ECF) space to support digestive processes in the intestinal lumen (22) (enterosystemic cycle; see below). Because 30–55% of daily water loss is accounted for in feces, horses off feed defecate less and require less water than fed horses with normal fecal output (6).

The effects of feed deprivation vs. feeding on voluntary water consumption was studied in eight healthy adult Thoroughbred geldings in a randomized crossover design, with each horse serving as its own control (fed conditions) (11). Feed deprivation reduced water consumption to ~16% of fed values immediately after feed was denied, and this persisted with evidence of mild dehydration only on day 4 (11). When unmeasured variables are considered, such as water from feed and metabolic water (9), the difference in total water intake between feed deprivation (10.3 ml/kg/day) and the fed state (71.4 ml/kg/day) was even greater than indicated by voluntary water consumption only (11).

Water consumption in ponies denied feed in another study decreased to 27% the volume consumed when they were fed (29). Because water turnover is directly related to metabolic weight (30), water needs established for ponies are probably unsuitable for large-breed horses (10). The diarrhea that developed in three of eight horses toward the end of a 4-day interval without feed (11) is difficult to explain and could possibly be attributed to altered microbiota (31) and VFA production (32) or by alteration of the enterosystemic cycle when digestive processes are interrupted.

## Enterosystemic Cycle

**Figure 1:**
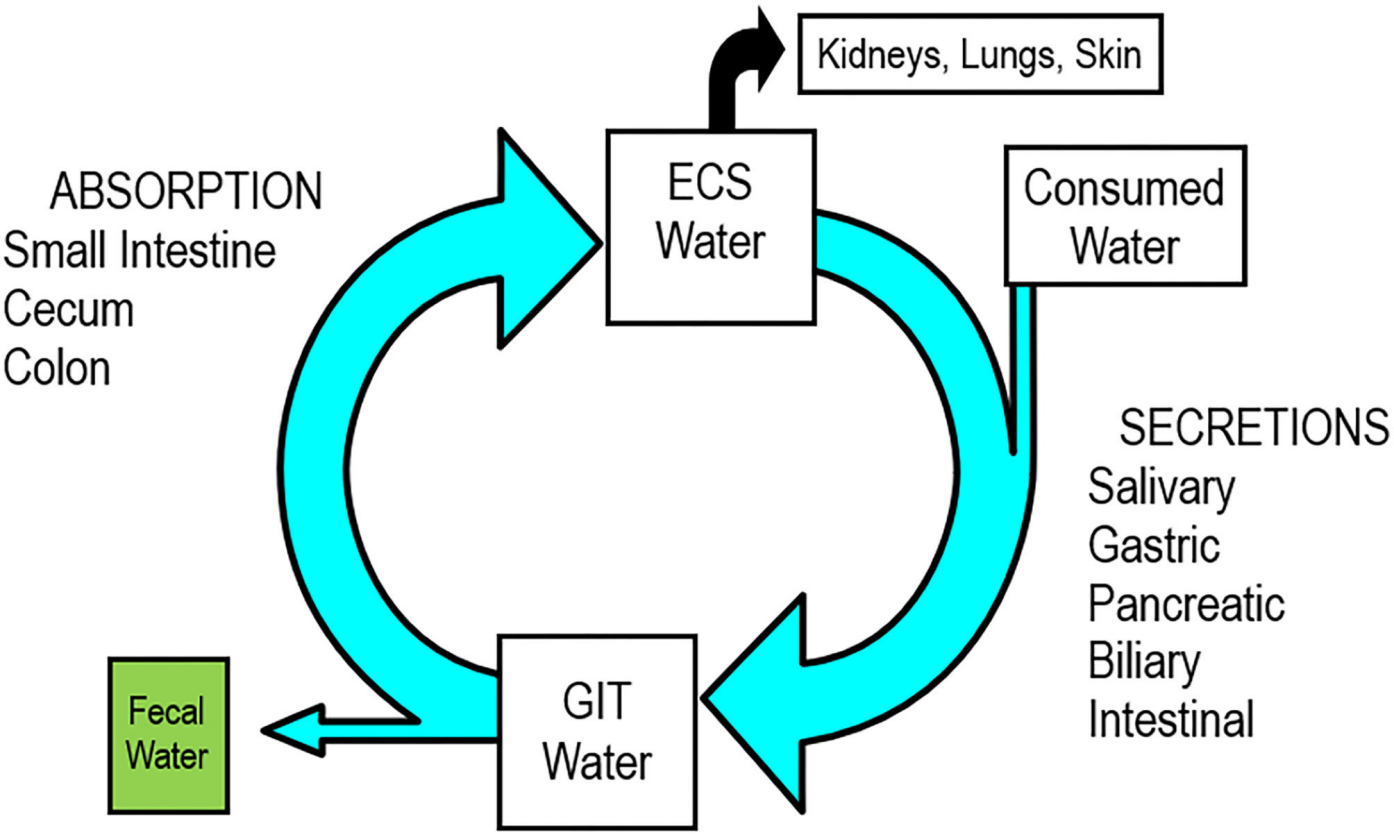
Enterosystemic cycle in the healthy horse, to demonstrate the interplay between the gastrointestinal tract and the extracellular fluid (ECF) space. Absolute values are not included, which would identify the relative contributions of each process or segment to the overall scheme, although different sizes of arrows and boxes represent approximate relationships. Font sizes do not represent differences, such that the cecum and colon are responsible for greater water and electrolyte absorption than the small intestine.

The relationship between consumed water, the intestinal tract, and the horse's hydration status are demonstrated in the enterosystemic cycle (Figure 1). The enterosystemic cycle describes how ECF water is used to support enzymatic and microbial digestion and is then reclaimed during the interdigestive period to preserve the ECF volume (22, 33) (Figure 1). This water movement is characterized by periods of net influx and efflux, driven by a cyclic pattern of microbial digestion in response to feed intake (33). This relationship between the ECF and gut also plays a critical role in survival in an arid environment by preserving systemic hydration through water absorption from a hindgut reservoir that seems anatomically and physiologically well-suited for that purpose (4, 5, 8, 34).

Horses drink predominantly in the early postprandial period (11, 35), presumably to correct dehydration induced by influx of extracellular water into the gastrointestinal tract (22) in response to the meal-induced increases in osmotic load (3) and to support enzymatic and microbial digestion (33). This high volume of water influx into the intestine in response to digestion can induce a 15% reduction in plasma volume, sufficient to activate the renin–angiotensin–aldosterone system. This response could contribute to water reabsorption (22) (Figure 1). Aldosterone can double Na^+^ and presumably water reabsorption by the ventral and dorsal colons and can triple these responses in the small colon (36).

Most of the water horses consume quickly reaches the large intestine, either through rapid and direct transit (33) or through small intestinal absorption followed by colonic secretion (8). This consumed water contributes to a substantial reservoir of water and electrolytes in the gastrointestinal tract, with 74% to 81% in the large intestine, and the remainder in the stomach and small intestine (37). Water constitutes more than 80% of intestinal contents (37), which means that a horse with a digestive tract capacity of 120 to 150 L has a water reservoir of >100 L in the intestinal tract (5). Because colon contents account for 13% body weight (38), as much as 50 L of water per 500-kg body weight can be contained in this segment (4, 9). Daily water reclamation from the colon to preserve the ECF requires reabsorption of the equivalent of >95% of the Na^+^, ${\text{HCO}}_{3}^{-}$, and water in the animal's ECF volume (33).

The enterosystemic cycle presumably undergoes lower rates of water exchange in horses managed by high-frequency meal feeding programs that are more natural than the typical twice a day feeding of hay and grain (22). Possibly, the natural grazing system preserves the ECF water more efficiently, which could explain why feral horses can travel up to 55 km from water and thereby tolerate large intervals between watering (39). The lack of feed from overgrazing close to the water source (39) could reduce feed intake in the feral horse as its water needs increase, thereby reducing demand on ECF water to support digestive processes when water is scarce.

## The Role of Thirst

Water consumption is driven by internal cues prompted by dehydration and hypovolemia (40) and combines with water absorbed from the colon (4) if needed as an auxiliary source. Many osmotic, ionic, hormonal, and neural signals are integrated within the central nervous system to stimulate water consumption (40). Water consumption is positively correlated with plasma osmolality in horses (3, 8, 40, 41), which is accounted for mostly by plasma Na^+^ (8). Plasma Na^+^ can decrease in horses during feed deprivation (11), probably from reduced intake and increased urinary loss (5). Feed deprivation, with (5, 26) or without water deprivation (11), can induce diarrhea in horses, which could also increase fecal loss of Na^+^ (42). Horses allowed to eat hay but denied water will develop a free water deficit, with increased serum Na^+^ concentration, serum osmolality, and thirst (8).

The response to thirst in horses can rapidly correct dehydration from water deprivation or water loss and effectively restore plasma osmotic pressure and blood volume (3). A 3% increase in osmolality or 8 mOsm increase will stimulate thirst, which is similar to other animals (3). Even minor cellular dehydration is a powerful stimulus to osmoreceptive neurons in the preoptic/hypothalamic region of the brain (40) and would be expected to stimulate thirst in horses (43). Horses will drink in response to an isosmotic loss of blood volume of 6% (3), which is considerably less than the 15% loss in plasma volume recorded in horses within 1 h after they start a large meal. This could explain the role of voluntary postprandial drinking to restoring early water loss during meal feeding. However, “involuntary dehydration” has been reported in endurance horses, possibly by an unknown mechanism that could dampen the thirst drive (12). Ponies that are deprived of water can also overcompensate by drinking more than would be expected, presumably to restore the water reservoir in the colon (3). During initial rehydration after a period of water deprivation, most of the consumed water in equids accumulates in the hindgut (4), either through direct passage or small intestinal absorption followed by secretion into the hindgut (8).

Peripheral or visceral osmoreceptors can also modify water consumption (44). Visceral osmoreceptors are located in the upper parts of the alimentary tract, oropharyngeal cavity, gastrointestinal tract, and liver (44). These detect consumed dilute fluids and preemptively signal the inhibition of vasopressin release (44).

## Replacement Fluid Therapy

Fluid therapy in horses is typically designed to correct fluid deficits from diarrhea, gastrointestinal reflux, or prolonged sweating or to support altered hemodynamic status with maldistributive shock. It is guided in veterinary medicine by crude clinical and laboratory indicators of efficacy (2). Such therapy also provides for maintenance needs (45), and in fact, resuscitative strategies for horses with postoperative reflux, a well-known source of copious fluid loss, typically include maintenance delivery rates as the standard approach in most hospitals (46, 47). Because these horses are not eating, they are actually receiving multiples of maintenance delivery rates, based on the 16% reduced water needs compared with fed values. This approach is probably of little consequence if it offsets fluid losses as intended, especially in these horses that are so dependent on fluid therapy to offset intestinal losses and to preserve renal function. However, a more conservative approach guided by meaningful measures of fluid responsiveness would probably benefit many of these cases and avoid any risk of overhydration (48–51). A heavy reliance on traditional maintenance rates in horses without a measure of fluid responsiveness could lead to overhydration, especially during anesthesia, when horses with colic can receive up to 15–25 ml/kg/h, with a mean of 19.5 ml/kg/h in one study (48).

Awareness of problems with overly aggressive fluid therapy in human patients and small animals and current concepts about fluid responsiveness (2, 49–51) could be relevant to horses. Excessive administration of Na^+^-rich crystalloids could be potentially harmful in human patients (49) and could reduce colloid osmotic pressure to levels associated with decreased survival in horses (52). A low intraoperative PCV in horses could predict failure to recover from anesthesia and the need for postoperative gastric decompression (53), possibly as an adverse response to overhydration. Although horses have developed efficient homeostatic mechanisms for dealing with hypovolemia, a consequence of evolving to survive in arid environments (4, 34), they might not be equipped to manage a largely iatrogenic and unnatural phenomenon such as volume overload (51).

Volume overload can release natriuretic peptides from myocytes in response to increased cardiac filling pressures, and these peptides cleave membrane-bound proteoglycans and glycoproteins from the endothelial glycocalyx (EGL) (51, 54). This complication is exacerbated by existing endothelial damage in patients with sepsis, leading to a rapid shift of intravascular fluid into the interstitial space with tissue edema (51) and impaired lymphatic drainage (54).

The intestine seems to be more prone to edema formation than the lungs and skeletal muscle (55), possibly because of its relatively large extracellular space compartment (56). In a murine model of postoperative ileus (POI), high volume resuscitation and mesenteric venous hypertension caused significant intestinal edema and decreased small intestinal transit (57). Intestinal edema alone can initiate or propagate dysfunctional signaling pathways that disrupt intestinal contractility, even in the absence of neutrophil inflammation and mucosal injury (57, 58). This problem could be self-perpetuating, because lymph flow from intestine depends on peristalsis, so reduced lymphatic outflow in hypomotile intestine could exacerbate the intestinal edema (57, 58). Increased capillary permeability from shock and ischemia/reperfusion injury could also contribute to fluid leakage into the interstitium (57) and further impair gastrointestinal function following crystalloid infusions. Intestinal wall edema can also cause translocation of endotoxin or bacteria (50), increase intraabdominal hypertension (IAH), and even cause abdominal compartment syndrome (59).

## Maintenance Fluid Therapy

When the need for replacement fluid therapy has ended in the hospitalized horse, and it is considered no longer in shock or dehydrated, but is not being fed, then maintenance fluid at conservative rates can be given. Typical examples are horses with medically responsive colic, horses that have restricted access to feed and water for any reason, and horses with botulism or other causes of dysphagia, resolving colitis, or because recovery of alimentary tract function is incomplete (6) (oral, pharyngeal, esophageal injury, or gastrointestinal disease). Although voluntary water consumption would be ideal in such cases, concerns about function of the alimentary tract could necessitate IV infusions.

Maintenance fluids for IV administration, such as the commercially available “half strength” saline with dextrose (0.45% NaCl and 2.5% or 5% dextrose in water), can provide free water through dextrose metabolism (6). Dextrose-containing solutions could also be of benefit to miniature horses, ponies, and donkeys, because these are prone to hyperlipidemia, and to pregnant and lactating mares, because of their considerable energy needs (20). Some maintenance solutions lack K^+^, other electrolytes, and alkalinizing agents, and others provide a higher concentration of K^+^ than replacement fluids (6). The equal concentrations of Na^+^ and Cl^−^ (77 mEq/L) in these fluids produce a strong ion difference (SID) of 0, which could lead to hyperchloremic metabolic acidosis if given in high volumes (54). Hyponatremia is a possible but unreported complication with hypotonic maintenance fluids in horses (6). Although clinical and laboratory assessment of response to fluid therapy should be applied to horses on maintenance fluids, maintenance of a normal central venous pressure (CVP) could also be considered a reasonable goal to prevent edema, with high normal CVP regarded as the acceptable upper limit (6). However, this is a technically difficult measurement and of questionable value for measuring intravascular volume (51).

The critical problem with current maintenance fluid therapy is that the appropriate fluids are not available in the volumes perceived as necessary to meet maintenance needs of horses (60), and consequently, commercial replacement/resuscitation fluids in 5-L bags are used instead. However, these fluids are designed to replace water and electrolyte losses on an equivalent basis to plasma and are therefore too rich in Na^+^ for maintenance needs (8, 14, 60). In horses, an all-hay diet without salt supplement would provide 329 to 440 mEq of Na^+^ daily (9), less than the Na^+^ contained in 3 L of a commercially available balanced electrolyte solution (133 to 140 mEq/L). Therefore, a horse supplemented with a replacement electrolyte solution infused at the currently proposed maintenance rate (60 ml/kg/day) would receive almost 10 times its normal daily intake of Na^+^. Such Na^+^-rich fluids can induce sufficient diuresis to increase urinary losses of K^+^, Ca^++^ and Mg^++^ (8, 60–62). A balanced electrolyte solution infused IV at one to three times the current maintenance rate can cause net secretion of Na^+^ into the intestinal tract, quadruple fecal Na^+^ output, and decrease serum Na^+^ concentration in water-deprived horses (8). The Na^+^ influx into the gastrointestinal tract would entrain an influx of water with it to preserve osmotic equilibrium, and such water movement could cause dehydration (8, 14).

The preceding concerns can be resolved by infusing maintenance-purpose fluids in rates more appropriate for horses on feed restriction, which is about 16% of the current maintenance rates determined for fed horses (11) (~10 ml/kg/day). Such rates of infusion would also reduce the difficulties with currently available maintenance fluids, such as availability in small volumes, excessive dextrose infusion, high cost, and possibilities of hyponatremia and overhydration. However, further studies are required to demonstrate such benefits.

## Conclusions

Fluids constitute one of the single most expensive components of the total cost of colic treatment in a hospital setting, for both medical and surgical cases (63), and their use should be guided by an understanding of the many variables that underlie water balance in the horse. Recent research findings should increase awareness in horse owners and veterinarians that water consumption in equids can be considerably altered by feed consumption (11). Further research is needed to explore the many variables relevant to rehydration of sick horses and maintenance needs under different clinical conditions.

## Author Contributions

DF is the sole author, responsible for all parts of manuscript generation.

## Conflict of Interest

The author declares that the research was conducted in the absence of any commercial or financial relationships that could be construed as a potential conflict of interest.
